# Cutaneous manifestations in stroke patients and their association with functional rehabilitation parameters

**DOI:** 10.1007/s11845-026-04423-x

**Published:** 2026-05-02

**Authors:** Nihal Sarı, Zerrin Kasap, Benay Keleş, Işıl Deniz Oğuz, Sevgi Kulaklı, İlker Fatih Sarı

**Affiliations:** 1 The Department of Dermatology and Venereology, Giresun University Faculty of Medicine, Giresun, Turkey; 2 The Department of Physical Medicine and Rehabilitation, Giresun University Faculty of Medicine, Giresun, Turkey; 3 The Department of Physical Medicine and Rehabilitation, Rize State Hospital, Rize, Turkey; 4 Department of Dermatology and Venereology, İstanbul Medipol Mega University Hospital, İstanbul, Turkey; 5 The Department of Data Science and Analytics, Giresun University Faculty of Arts and Science, Giresun, Turkey

**Keywords:** Functional status, Fungal infections, Onychomycosis, Rehabilitation, Skin diseases, Stroke

## Abstract

**Background:**

Stroke is a focal neurological deficit resulting from vascular lesions of the brain. Stroke-related sequale, including poor hygiene, inadequate positioning, and insufficient skin care are associated with the development of dermatological problems.

**Aims:**

To evaluate the prevalence of cutaneous findings in stroke patients and their association with clinical characteristics and functional outcomes.

**Methods:**

This cross-sectional study included consecutive patients aged ≥18 years with a history of stroke for at least 3 months. Clinical status was assessed using the Brunnstrom Recovery Stage (BRS) and Standardized Mini-Mental State Examination (S-MMSE), and functional outcomes using the Functional Ambulation Scale (FAS) and Barthel Index of Activities of Daily Living (Barthel ADL Index).

**Results:**

A total of 104 stroke patients were enrolled. The most common cutaneous findings were xerosis (76.9%), skin infections (73.1%), nail changes (71.2), hair changes (51.9%), primary dermatoses (46.2%), and pruritus (42.3%). Skin infections were observed in 76 patients (73.1%), with fungal infections being the most frequent, including tinea pedis (58.7%),tinea unguium (50%). Patients with fungal infections had lower FAS scores (2.26±1.68 vs. 2.97±1.64; *p* = 0.042) and shorter stroke duration (median 11vs.36 months; *p* = 0.034). Patients with onychomycosis also had significantly lower FAS, Barthel ADL Index, and S-MMSE scores (all *p* < 0.05).

**Conclusions:**

Xerosis, skin infections, nail changes were identified as the most frequent cutaneous findings. Lower functional and cognitive scores were associated with fungal infections. Integrating dermatological care and hygiene protocols into stroke rehabilitation may improve care for patients with limited mobility and cognitive impairment; however longitudinal studies are needed to clarify these associations.

## Introduction

In embryonic development, the skin and the nervous system originate from the ectoderm, highlighting their close developmental relationship. This shared embryonic origin suggests a potential association between dermatological and neurological disorders [1]. In a study by Tanburoğlu et al., dermatology consultations were evaluated for neurological patients hospitalized in both the neurology intensive care unit and the neurology ward within a tertiary care hospital system. The study found that infectious dermatoses were the most frequent dermatological findings in patients hospitalized in the neurology clinic. Following this, drug eruptions and physical dermatoses were the second most frequently observed conditions, while eczematous disorders ranked third in prevalence. It has been reported that 39.6% of skin lesions develop during hospitalization [2]. In this context, dermatological complications appear to occur more frequently in neurological patients with prolonged hospitalization.

Stroke is a focal neurological deficit that results from impaired brain function due to various vascular lesions [3]. In clinical practice, stroke is diagnosed and treated in the acute phase within neurology clinics, whereas the chronic phase is primarily managed through long-term rehabilitation programs under the supervision of physical medicine and rehabilitation specialists. Hygiene deficiencies, improper positioning, and inadequate skin care resulting from stroke-related sequelae may predispose patients to dermatological complications. From this perspective, it is recommended that patients undergo regular evaluation for dermatological problems and that preventive measures be implemented to maintain skin integrity [4]. It is assumed that identifying and understanding the skin problems commonly observed in stroke patients may facilitate the development of appropriate prophylactic skin care strategies during the rehabilitation process. The literature to date lacks studies specifically investigating cutaneous manifestations in patients with stroke. Because dermatological problems in the early post-stroke period may be influenced by acute hospitalization and transient care-related factors, the chronic rehabilitation phase (> 3 months after stroke onset) was selected as the target population. The primary objective was to evaluate the prevalence of cutaneous manifestations in these patients, hypothesizing that their presence and burden may be associated with rehabilitation parameters—including ambulation level, functional independence, cognitive status, and motor recovery.

## Materials and methods

This cross-sectional study was conducted at Giresun Training and Research Hospital between February 2025 and August 2025. Due to the lack of prior comprehensive data on cutaneous manifestations in this specific patient population, the study was designed as an exploratory cross-sectional analysis without an a prior sample size calculation. Instead, a time-bound consecutive sampling strategy was employed, and all eligible patients presenting during the study period were included to minimize selection bias. Consecutive patients aged 18 years and older, diagnosed with chronic stroke of more than 3 months’ duration, who were admitted to the outpatient or inpatient services of the Physical Medicine and Rehabilitation Clinic and provided informed consent, were enrolled in the study. Patient demographics, stroke duration, stroke type (ischemic/hemorrhagic), and hemiplegic side (right/left) were recorded. The primary outcome was the frequency of cutaneous manifestations, and secondary outcomes were their associations with clinical and rehabilitation parameters (Brunnstrom Recovery Stage (BRS), Functional Ambulation Scale (FAS), Barthel Index of Activities of Daily Living (Barthel ADL index), and the Standardized Mini-Mental State Examination (S-MMSE). All patients underwent a comprehensive dermatological examination performed independently by two dermatologists under daylight conditions, including evaluation of the skin, hair, scalp, oral and genital mucosa, and nails. To improve diagnostic accuracy and methodological reliability, all dermatological findings were cross-validated by both dermatologists. When clinically indicated, native preparation, Wood’s lamp examination, dermoscopic evaluation, and/or skin biopsy were performed to support the diagnosis. In cases of diagnostic uncertainty, the final diagnosis was established by consensus between the two dermatologists. Photographic documentation of dermatological findings was performed at appropriate anatomical sites following the acquisition of informed patient consent. A neuromusculoskeletal examination was also performed by a physical medicine and rehabilitation specialist and, stroke patients were comprehensively evaluated using the BRS, FAS, Barthel ADL index, and S-MMSE.

The BRS was used to evaluate motor function recovery after stroke. In this system, stage 1 represents flaccidity with absence of voluntary movement, whereas stage 6 denotes the ability to perform isolated joint movements, reflecting the highest level of recovery [5]. Ambulation ability was assessed using FAS, which classifies patients into six stages (0–5) based on the motor skills required for walking. Higher scores indicate greater ambulation capacity and functional independence [6]. The Barthel ADL Index was utilized to assess functional independence across ten core daily self-care activities, with total scores ranging from 0 to 100 to categorize patients from total dependency to full independence [7]. Cognitive status was assessed using the S-MMSE, a 30-point test evaluating orientation, registration, attention/calculation, recall, and language [8]. A Turkish validity and reliability study was conducted by Gürgen et al. [9]. The study was conducted in accordance with the ethical principles of the Declaration of Helsinki. Before initiating the study, institutional ethics committee approval numbered 8.01.2025/11 was obtained from the Scientific Research Ethics Committee of Giresun Training and Research Hospital.

### Statistical analysis

Statistical analyses were performed using the SPSS 24.0 package program. Categorical variables were presented as numbers and percentages (n, %). There were no missing data for dermatologic outcomes and core rehabilitation measures; therefore, all analyses were performed on complete data.Normality of distribution for continuous variables was evaluated based on Skewness and Kurtosis values between − 1.5 and + 1.5, which were considered indicative of normal distribution. Continuous variables with normal distribution were presented as mean ± standard deviation (mean ± SD), whereas non-normally distributed data were expressed as median and interquartile range (IQR). For comparisons between two independent groups, the Student’s t-test was applied when the assumption of normality was met, while the Mann-Whitney U test was used otherwise. A p-value < 0.05 was accepted as statistically significant for all analyses.

## Results

**Fig. 1:**
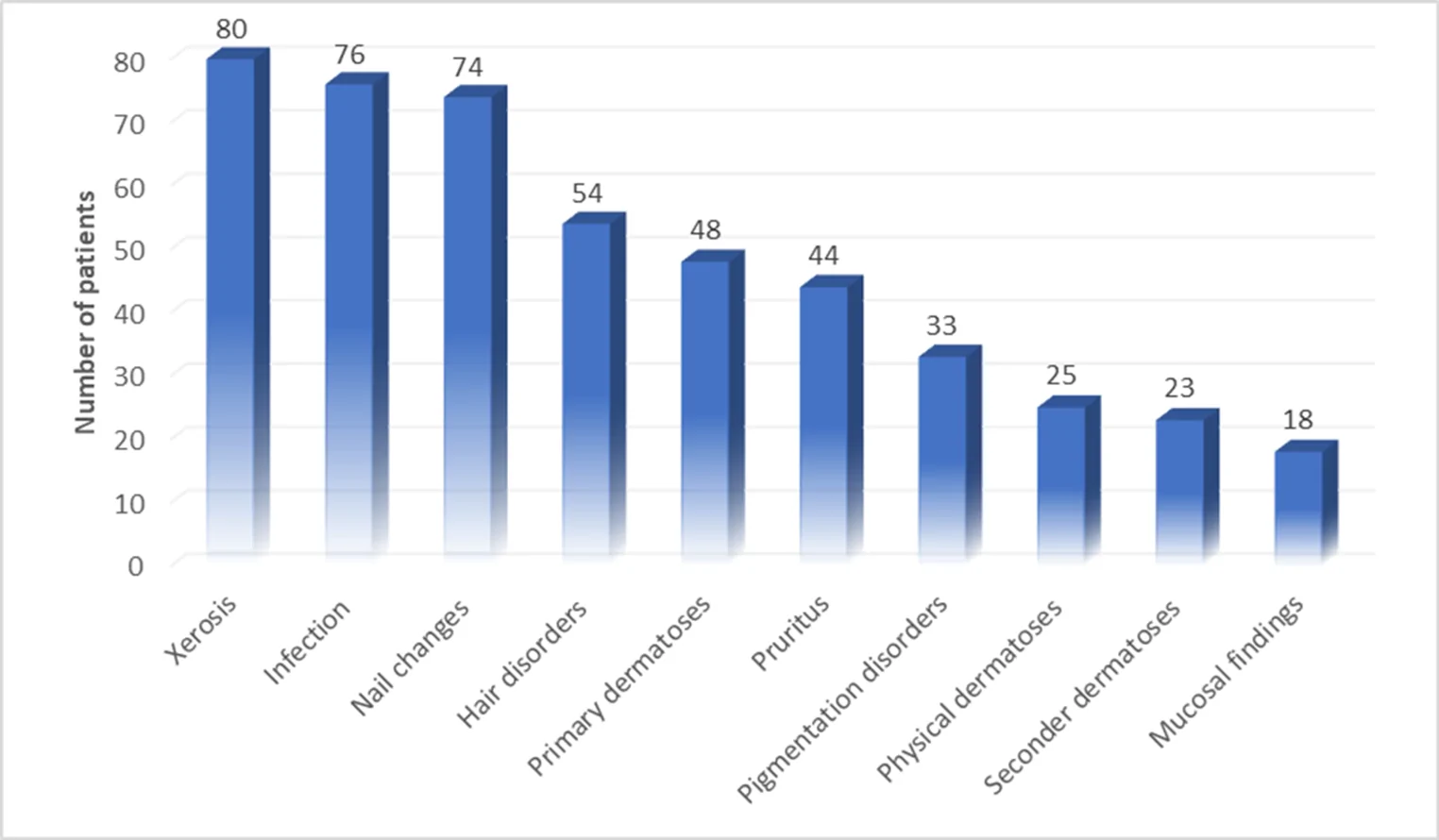
Distribution of skin findings in stroke patients

**Fig. 2:**
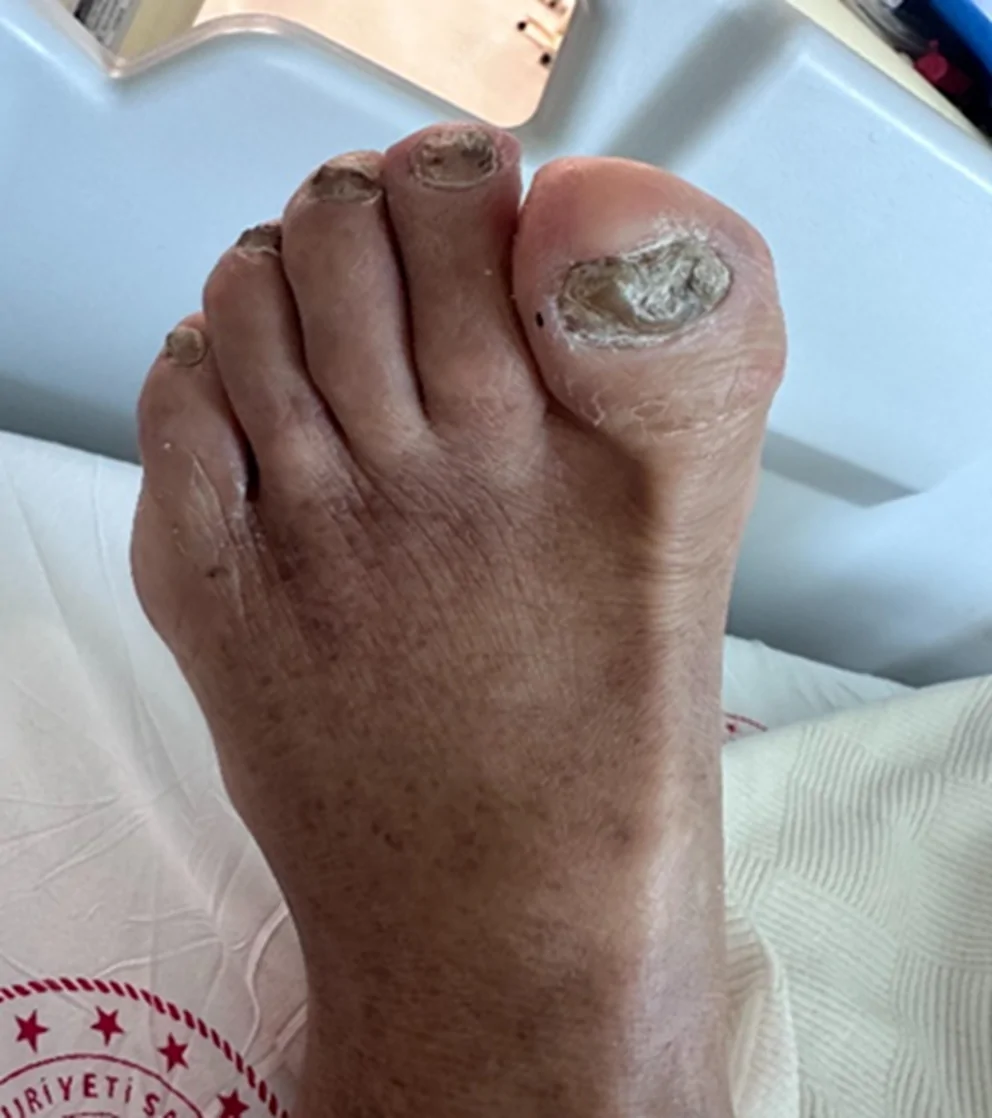
Clinical image of Tinea pedis and unguium in a stroke patient

A total of 104 stroke patients were included in the study, comprising 62 females (59.6%) and 42 males (40.4%). Regarding stroke etiology, 90 patients (86.5%) had ischemic stroke, while 14 (13.5%) had hemorrhagic stroke. The mean age of the patients was 65.13 ± 13.81 years. The demographic characteristics, motor recovery status, functional ambulation level, degree of independence in daily living activities, and cognitive status of the stroke patients are presented in Table 1. Evaluation of cutaneous manifestations in stroke patients revealed that xerosis (76.9%, *n* = 80) was the most common finding, followed by skin infections (73.1%, *n* = 76), nail changes (71.2%,*n* = 74), hair disorders (51.9%, *n* = 54), primary dermatoses (46.2%, *n* = 48), and pruritus (42.3%, *n* = 44) (Fig. 1). Among infectious skin conditions, fungal infections were the most frequent. Tinea pedis was present in 61 patients (58.7%), and tinea unguium was present in 52 patients (50%) (Table 2). The clinical picture of tinea pedis and tinea unguium in a stroke patient is presented in Fig. 2. Among primary dermatoses, seborrheic dermatitis was the most common (25.0%, *n* = 26). Among secondary dermatoses, pruritus-related excoriations were the most frequent (21.2%, *n* = 22). Among physical dermatoses, pressure injury was seen in 4.8% (*n* = 5) and callus was seen in 1.9% (*n* = 2).

**Table 1:** Demographic and clinical characteristics of patients

Sex	
Female	42 (40.4%)
Male	62 (59.6%)
Age, years	65.13±13.81
Etiology	
Ischemic	90 (86.5%)
Hemorrhagic	14(13.5%)
Hemiplegic side	
Right	53 (51.0%)
Left	48 (46.2%)
Bilateral	3 (2.9%)
Brunstroom Recovery Stage	
Arm	3.78±1.68
Hand	3.74±1.67
Lower extremity	3.99±1.46
FAS	2.5±1.69
FAS	
0	22 (21.2%)
1	9 (8.7%)
2	17 (16.3%)
3	17 (16.3 %)
4	29 (27.9%)
5	10 (9.6%)
Barthel Activities of Daily Living Index	57.34±31.56
S-MMSE	19.57±8.92

*FAS,* Functional Ambulation Scale; *Barthel ADL Index,* Barthel Activities of Daily Living Index; *S-MMSE,* Standardized Mini-Mental State Examination

**Table 2:** Distribution of stroke patients according to skin findings

	Number of patients (%)
*Xerosis*	80 (76.9 %)
*Pruritus*	44 (42.3 %)
*Skin infections*	76 (73.1 %)
Bacterial infections	11 (10.6 %)
Intertrigo	2 ( 1.9 %)
Folliculitis	9 (8.7 %)
Fungal infections	69 (66.3 %)
Tinea pedis	61 (58.7 %)
Tinea Unguium	52 (50.0 %)
Tinea Cruris	9 (8.7 %)
Other*	8 (7.7 %)
Parasitic infections	8 (7.7 %)
Scabies	6 (5.8 %)
Demodicosis	2 (1.9 %)
Viral infections	4 ( 3.8 %)
*Primary Dermatoses*	48 (46.2 %)
Seborrheic Dermatitis	26 (25.0 %)
Rosacea	16 (15.4 %)
Contact-dyshidrotic dermatitis	7 ( 6.7 %)
Acne	4 ( 3.8 %)
Psoriasis	2 ( 1.9 %)
*Secondary Dermatoses*	23 (22.1 %)
Excoriations	22 (21.2 %)
Diabetic dermopathy	2 (1.9 %)
Pseudoporphyria	1 (1 %)
*Physical Dermatoses*	25 (24 %)
Hypertrophic-Keloid Scar	15 (14.4 %)
Hyperkeratosis	9 ( 8.7 %)
Pressure injury	5 (4.8 %)
Stria	2 ( 1.9 %)
Callus	2 ( 1.9%)

The frequency of hair, nail, mucosal findings and skin pigment changes of the patients is presented in Table 3. Among these findings, nail alterations were the most frequent, observed in 74 patients (71.2%). The predominant nail changes included subungual hyperkeratosis (52.9%), onychomycosis (50%), and longitudinal ridges (20.2%).

**Table 3:** Pigmentation, hair, nail and mucosal findings in patients

	Number of patients (%)
Mucosal findings*	18 (17.3%)
*Hair disorders*	54 (51.9 %)
Androgenetic alopecia	54 (51.9 %)
Oily hair	3 ( 2.9 %)
Hypertrichosis-hirsutism	2 ( 1.9 %)
Thin Weak hair	1 ( 1.0 %)
*Nail changes*	74 (71.2 %)
Subungual hyperkeratosis	55 (52.9 %)
Onychomycosis	52 (50.0 %)
Longitudinal ridges	21 (20.2 %)
Absence of lunula	5 ( 4.8 %)
Onycholysis	5 ( 4.8 %)
*Pigmentation Disorders*	33 (31.7 %)
Ecchymosis	16 (15.4 %)
Melasma	9 ( 8.7 %)
Postinflammatory hyperpigmentation	6 ( 5.8 %)
Hypopigmentation	3 ( 2.9 %)

*Fissured/geographic tongue, aphthous stomatitis

Clinical parameters were compared between patients with and without each cutaneous finding. No significant differences were found for xerosis, nail changes, hair disorders, pruritus, physical dermatoses, or secondary dermatoses in terms of arm, hand, and upper extremity BRS scores, FAS scores, stroke duration, or S-MMSE scores (all *p* > 0.05).FAS scores were significantly higher in patients without skin infection compared to those with infection(3.11 ± 1.55 vs. 2.28 ± 1.69; *p* = 0.025). Additionally, the duration of stroke was significantly shorter in patients with skin infection than in those without skin infection (median: 12 months, IQR: 32.50 vs. median: 36 months, IQR: 85.25; *p* = 0.021). However, no significant differences were observed between the groups with respect to BRS, Barthel ADL Index, and S-MMSE scores in terms of duration of stroke (all *p* > 0.05).

When skin infection subgroups were analyzed, FAS scores were found to be significantly higher in patients without fungal skin infection compared to patients with fungal infection (2.97 ± 1.64 vs. 2.26 ± 1.68; *p* = 0.042). The duration of the stroke was significantly longer in patients without fungal skin infection (median: 36.00 months, IQR: 62.00) than in those with fungal skin infection (median: 11.00 months, IQR: 32.00; *p* = 0.034). No statistically significant differences were observed between the groups in terms of other parameters (BRS, Barthel ADL Index, and S-MMSE scores) (all *p* > 0.05).

**Fig. 3:**
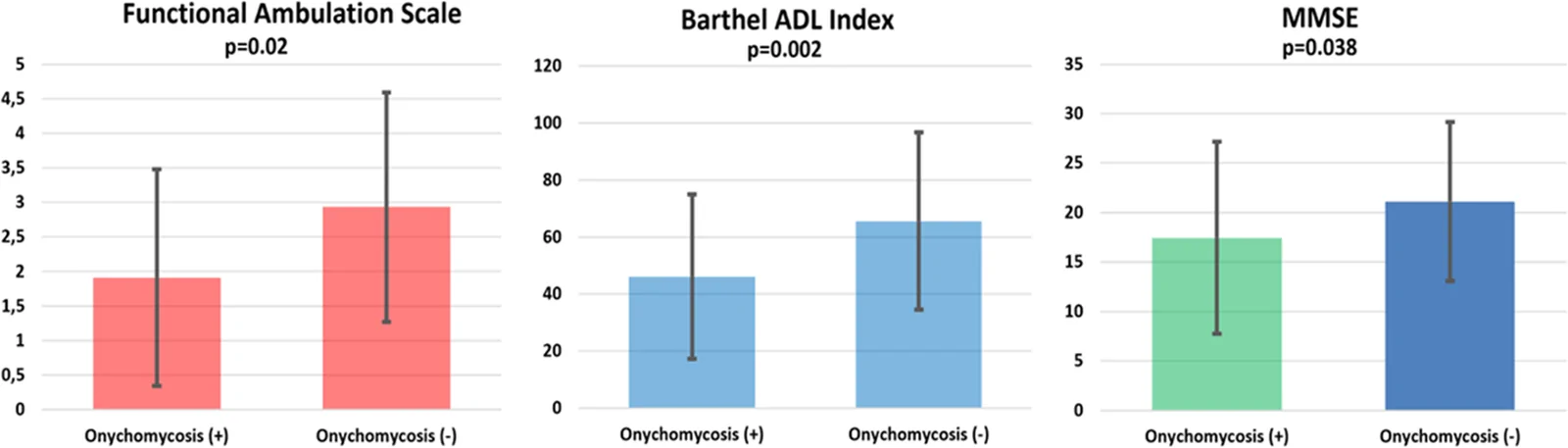
Comparison of functional independence, self-care, and cognitive status according to the presence of onychomycosis

**Fig. 4:**
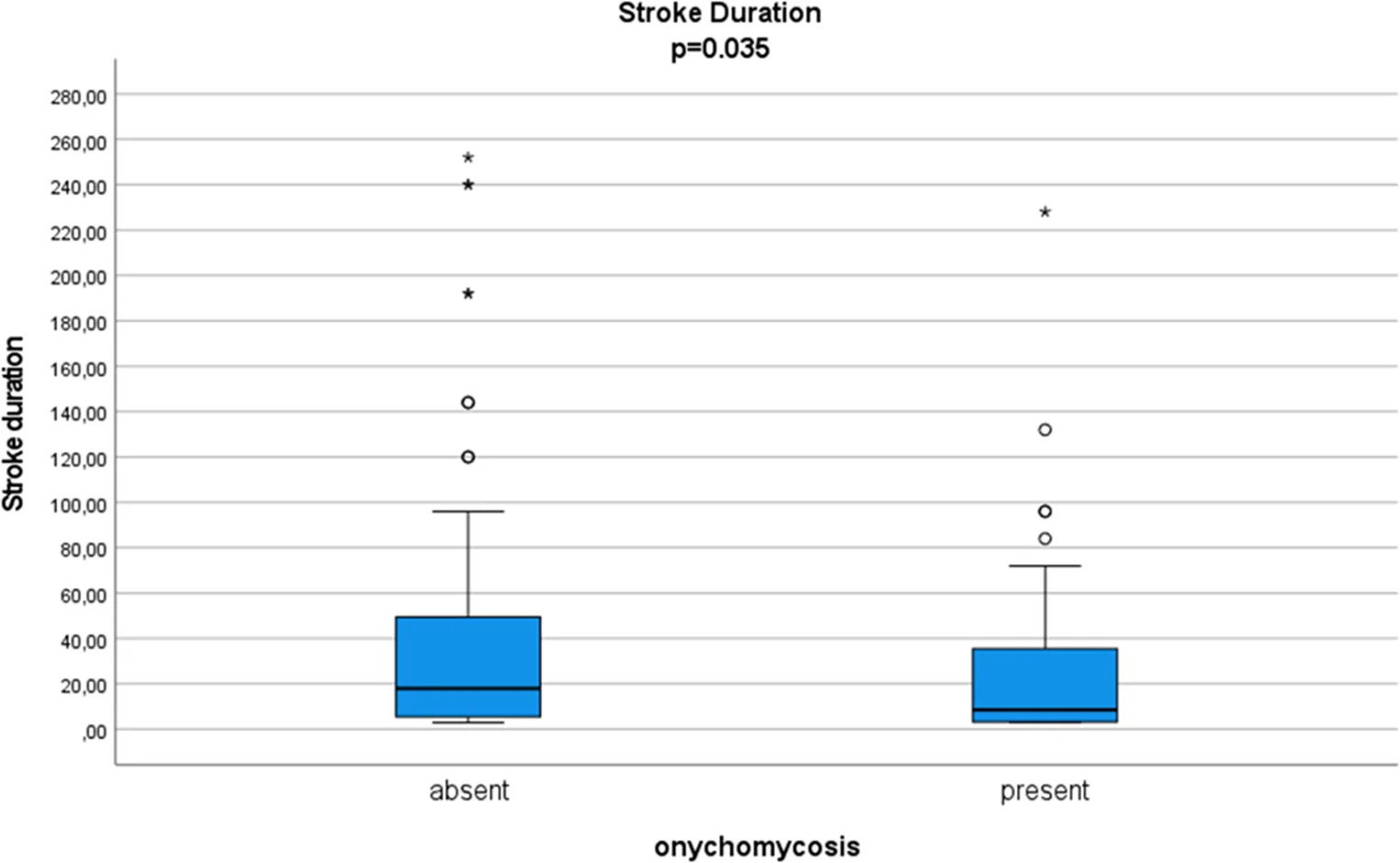
Comparison of stroke duration in patients with and without onychomycosis

When onychomycosis was analyzed separately, FAS scores were significantly higher in patients without onychomycosis than in those with onychomycosis (2.93 ± 1.66 vs. 1.91 ± 1.57, *p* = 0.020). Similarly, Barthel ADL Index scores (65.58 ± 31.11 vs. 46.09 ± 28.87; *p* = 0.002) and S-MMSE scores (21.12 ± 8.04 vs. 17.45 ± 9.69; *p* = 0.038) were found to be significantly higher in patients without onychomycosis (Fig. 3). Additionally, the duration of stroke was found to be statistically significantly higher in patients without onychomycosis compared to those with onychomycosis (median: 18 months, IQR: 45.00 vs. median: 8.5 months, IQR: 32.63; *p* = 0.035) (Fig. 4).

## Discussion

This study investigated the prevalence of dermatological findings in patients with stroke and explored their associations with disease-related parameters and functional outcomes. The most frequently observed manifestations were included xerosis, cutaneous infections, and nail changes. To the best of our knowledge, few studies have examined cutaneous problems in stroke patients from a broad dermatological perspective. The findings suggest that dermatological complications in chronic stroke patients may be associated with lower functional independence, and immobility-related factors.

All 104 stroke patients included in this study were found to have included in this study presented with at least one concomitant dermatological condition. The most frequent manifestations were xerosis, skin infections, nail changes, hair disorders, primary dermatoses, and pruritus, with xerosis being the most prevalent. Xerosis has been reported with a prevalence of up to 85% in the general population and is known to affect nearly all individuals aged 70 years and older. This high prevalence is attributed to age-related alterations in the skin, including reduced epidermal thickness, decreased water and lipid content, diminished sebum secretion, and impaired sweating, ultimately leading to xerosis [10]. The mean age of patients in this study was approximately 65 years, and was observed particularly frequently in stroke patients at a rate of 80%. In stroke patients, deficits in muscle strength, coordination, and motor function on the affected side may further limit the ability to adequately moisturize the skin, which may be associated with the high frequency of xerosis observed. Pruritus, detected in 44% of patients, is also likely associated with xerotic skin. Neuropathic pruritus may additionally occur secondary to somatosensory system damage following stroke [11]. Excoriations due to scratching were identified in 21.2% of patients. The disruption of skin integrity associated with these excoriations may provide a portal for microbial entry, potentially contributing to infection risk.

In addition to xerosis, skin infections were another frequently encountered group of dermatological conditions. Among these, fungal skin infections are particularly notable and have been reported at relatively high rates in geriatric populations. According to the literature, the prevalence of fungal skin infections in elderly patients ranges between 10% and 15% [12–14]. A large-scale study from Taiwan including 16,924 geriatric patients aged ≥ 65 years reported a high prevalence of fungal infections of 38% [15]. Age-related thinning of the epidermis renders the skin more fragile and vulnerable to damage even from minor friction, creating a potential entry point for microorganisms [16]. Another study found that the development of onychomycosis or tinea pedis was significantly associated with type 2 diabetes mellitus, increasing age, and male gender [17]. In a study evaluating dermatology consultations in neurological patients, the most common dermatological diagnosis was also found to be fungal skin infections [18]. Tinea pedis is a superficial fungal infection that usually occurs in warm and humid conditions. Insufficient drying of the feet and poor foot hygiene—particularly due to motor dysfunction and self-care limitations following stroke—may be associated with an increased risk of fungal foot infections. The high prevalence of tinea pedis observed in our study (58.7%) may be explained by the combined effects of stroke-related functional disability and age- associated deterioration of the skin barrier. A key finding of this study was that stroke patients with fungal skin infections demonstrated significantly lower FAS scores than those without infections. Since higher FAS scores reflect greater independence and ambulatory capacity, these patients are more likely to maintain mobility, ensure adequate foot hygiene, and regularly change shoes and socks, thereby reducing the risk of fungal infection. Conversely, patients with lower FAS scores experience greater immobility, insufficient foot hygiene, and inadequate drying, which may be associated with a favorable microenvironment for fungal colonization. These findings underscore the importance of foot hygiene and preventive strategies against fungal infections in patients with impaired ambulation during the rehabilitation process.

In this study, stroke patients with skin and fungal infections had significantly shorter stroke event time compared to those without infections. In other words, the frequency of infections declined as the time elapsed since stroke onset increased. However, given the cross-sectional design, these findings should be interpreted as associations, and no inference can be made regarding causality or changes over time. One possible interpretation is that, in the early stages post-stroke period, both patients and their caregivers often lack adequate experience and knowledge regarding critical aspects of stroke rehabilitation—particularly in maintaining skin integrity, ensuring proper hygiene, and adopting preventive skin care practices. Over time, as patients and their relatives gain more knowledge and experience in patient care, preventive measures can be implemented more effectively.However, since caregiver education and experience were not directly measured in this study, this remains a potential explanation for future research. This may be related to the observed decrease in infection rates among individuals with longer post-stroke durations.

Regarding the prevalence of tinea unguium, fungal culture positivity was reported in 28.1% of individuals aged 60 years and older [7]. Similarly, a study conducted in Turkey among individuals aged ≥ 65 years found a tinea unguium prevalence of 38.5% [19]. The increased frequency of tinea unguium in older adults is thought to be attributable to age-related physiological changes, including reduced nail growth rate, impaired peripheral circulation, and a decline in immune system function. In addition, functional limitations such as diminished vision and reduced mobility may further contribute to the higher prevalence of fungal nail infections in this population [20]. In our study, the prevalence of onychomycosis in stroke patients was 50%, which is relatively high. A previous study evaluating nail changes in hemiplegic patients reported characteristic nail alterations such as longitudinal red striations, Neapolitan nails, and unilateral clubbing. That study also found comparable frequencies of onychomycosis in hemiplegic patients and controls (11% vs. 9%) [21]. In this study, onychomycosis was observed in approximately half of the chronic stroke patients. This high prevalence may be partially explained by the characteristically humid climate of Giresun province, which may promote fungal colonization and is likely associated with the concurrent prevalence of tinea pedis. These findings underscore the importance of implementing regular and appropriate foot care practices in stroke patients, as such interventions may help reduce the incidence of fungal infections, including tinea pedis and tinea unguium.

In this study, stroke patients with onychomycosis demonstrated significantly lower scores on the FAS, Barthel ADL Index, and S-MMSE compared to those without onychomycosis. These findings indicate that the presence of onychomycosis in stroke patients may be associated with reduced ambulation capacity, diminished functional independence, and impaired cognitive performance. Consistently, Lubeck et al., in a study evaluating the quality of life of individuals with onychomycosis, reported significantly lower mental status scores in affected patients [22]. Dermatological complications following stroke are frequently overshadowed by major neurological sequelae and therefore tend to receive limited clinical attention. However, our results underscore that the presence of onychomycosis in patients with stroke is not merely a dermatological issue but may represent an important clinical marker of mobility dependence, self-care impairment, and cognitive decline.

In this study, the prevalence of pressure injury was 4.8% in patients with chronic stroke lasting longer than three months. In comparison, another study reported a prevalence of 8.3% in stroke patients [23], while a study conducted in Turkey found pressure injuries in 6.9% (*n* = 123) of 1216 patients [24]. The relatively low prevalence observed in our cohort may be attributed to the early initiation of rehabilitation programs, routine implementation of positional changes, and the effective use of pressure-relieving prophylactic interventions at our center.

One of the major limitations of the study was its cross-sectional design. Consequently, it was not possible to determine precisely when and how the dermatological findings developed, or whether they were present prior to stroke onset. Furthermore, as this was an exploratory study in a field with limited prior data, in a priori sample size calculation was not performed; however, a consecutive sampling strategy was utilized to include all eligible patients during the study period to ensure a representative cohort. Second, as this was a single-center study conducted in a rehabilitation clinic, selection bias is possible and the findings may not be generalizable to all chronic stroke populations, particularly community-dwelling individuals not receiving rehabilitation services.Third, patients in the acute and subacute post-stroke phases were not included; therefore, the findings cannot be generalized to dermatological manifestations occurring within the first 3 months after stroke. Nevertheless, despite these limitations, this study is valuable as it represents the first investigation on this topic, offers unique insights from an interdisciplinary perspective, and highlights a significant gap in the existing literature. Future prospective longitudinal studies are warranted to provide more robust evidence regarding cutaneous manifestations in stroke patients.

In conclusion, this study, which identified common skin findings in stroke patients, has important implications for both dermatologists and physical medicine and rehabilitation specialists. Integrating skin and skin appendage care and hygiene protocols into the rehabilitation process will be beneficial for common problems encountered in stroke patients, such as dry skin, skin infections, and nail disorders. Our findings underscore the necessity of a multidisciplinary approach for the early recognition and prevention of dermatological complications that may adversely affect functionality and independence.

## References

[1] Kupetsky EA, Rincon F (2013) The prevalence of systemic diseases associated with dermatoses and stroke in the United States: a cross-sectional study. Dermatology 227(4):330–337. 10.1159/000354912 24247071

[2] Tanburoglu A, Özçelik S (2022) Dermatological Problems in a Neurology Clinic. Cureus 14(11):e31994. 10.7759/cureus.31994 36589203 PMC9797868

[3] Shahid J, Kashif A, Shahid MK (2023) A Comprehensive Review of Physical Therapy Interventions for Stroke Rehabilitation: Impairment-Based Approaches and Functional Goals. Brain Sci 13(5):717. 10.3390/brainsci13050717 37239189 PMC10216461

[4] Mead GE, Sposato LA, Sampaio Silva G, guidelines on behalf of the World Stroke Organization (2023) A systematic review and synthesis of global stroke. Int J stroke: official J Int Stroke Soc 18(5):499–531. 10.1177/17474930231156753 PMC10196933 36725717

[5] Brunnstrom S (1966) Motor testing procedures in hemiplegia: based on sequential recovery stages. Phys Ther 46(4):357–375. 10.1093/ptj/46.4.357 5907254

[6] Holden MK, Gill KM, Magliozzi MR (1986) Gait assessment for neurologically impaired patients. Standards for outcome assessment. Phys Ther 66(10):1530–1539. 10.1093/ptj/66.10.1530 3763704

[7] Yavuzer MG, Süldür N, Küçükdeveci A et al (2000) Türkiye’de nörorehabilitasyon hastalarının değerlendirilmesinde fonksiyonel bağımsızlık ölçeği ve Modifiye Barthel İndeksi’nin yeri. Romatoloji ve Tıbbi Rehabilitasyon Dergisi 11(1):26–31

[8] Folstein MF, Folstein SE, McHugh PR (1975) Mini-mental state. A practical method for grading the cognitive state of patients for the clinician. J Psychiatr Res 12(3):189–198. 10.1016/0022-3956(75)90026-6 1202204

[9] Güngen C (2002) Standardize Mini Mental Test’in Türk toplumunda Hafif Demans Tanısında Geçerlilik ve Güvenilirliği. Türk Psikiyatri Dergisi 13:273–281 12794644

[10] Farage MA, Miller KW, Berardesca E et al (2009) Clinical implications of aging skin: cutaneous disorders in the elderly. Am J Clin Dermatol 10(2):73–86. 10.2165/00128071-200910020-00001 19222248

[11] Kwatra SG, Kambala A, Dong X (2023) Neuropathic pruritus. J Allergy Clin Immunol 152(1):36–38. 10.1016/j.jaci.2023.04.006 37094766

[12] Kulaklı S (2024) Skin diseases in geriatric patients: one-year single center experience. Acıbadem Univ. Sağlık Bilimleri Dergisi 15(2). 10.31067/acusaglik.1312484

[13] Bilgili SG, Karadag AS, Ozkol HU et al (2012) The prevalence of skin diseases among the geriatric patients in Eastern Turkey. J Pak Med Assoc 62(6):535–539 22755334

[14] Yaldiz M (2019) Dermatological diseases in the geriatric age group: Retrospective analysis of 7092 patients. Geriatr Gerontol Int 19(7):582–585. 10.1111/ggi.13665 30950155

[15] Liao YH, Chen KH, Tseng MP et al (2001) Pattern of skin diseases in a geriatric patient group in Taiwan: a 7-year survey from the outpatient clinic of a university medical center. Dermatology 203(4):308–313. 10.1159/000051778 11752818

[16] Varade RS, Burkemper NM (2013) Cutaneous fungal infections in the elderly. Clin Geriatr Med 29(2):461–478. 10.1016/j.cger.2013.01.001 23571040

[17] Oz Y, Qoraan I, Oz A et al (2017) Prevalence and epidemiology of tinea pedis and toenail onychomycosis and antifungal susceptibility of the causative agents in patients with type 2 diabetes in Turkey. Int J Dermatol 56(1):68–74. 10.1111/ijd.13402 27667305

[18] Williams A, Bhatia A, Kanish B et al (2016) Pattern of Inpatient Dermatology Consultations in a Tertiary Care Centre from Northern India. J Clin Diagn research: JCDR 10(12):WC07–WC10. 10.7860/JCDR/2016/21182.8968 28208985 PMC5296558

[19] Bas Y, Kalkan G, Seçkin HY et al (2014) Geriatrik Hastalarda Dermatolojik Sorunlarin Analizi/Analysis of dermatologic problems in geriatric patients. Turk Dermatoloji Dergisi 8(2):95

[20] Gupta AK (2000) Onychomycosis in the elderly. Drugs Aging 16(6):397–407. 10.2165/00002512-200016060-00002 10939306

[21] Siragusa M, Schepis C, Cosentino FI et al (2001) Nail pathology in patients with hemiplegia. Br J Dermatol 144(3):557–560. 10.1046/j.1365-2133.2001.04083.x 11260014

[22] Lubeck DP, Patrick DL, McNulty P et al (1993) Quality of life of persons with onychomycosis. Quality of life research. Int J Qual life aspects Treat care rehabilitation 2(5):341–348. 10.1007/BF00449429 8136799

[23] Farid J, Amin R, Sheikh MA et al (2022) Prevalence and prediction of pressure ulcers in admitted stroke patients in a tertiary care hospital. J tissue viability 31(4):768–775. 10.1016/j.jtv.2022.07.010 35941057

[24] Bilir Kaya B (2019) Pressure ulcer rates of stroke patients in a public rehabilitation hospital and training rates of nurses for pressure ulcer. J Surg Med 3(7):512–514

